# Class solutions for SABR-VMAT for high-risk prostate cancer with and without elective nodal irradiation

**DOI:** 10.1186/s13014-016-0730-7

**Published:** 2016-11-24

**Authors:** Sarah O. S. Osman, Prakash Jeevanandam, Nithya Kanakavelu, Denise M. Irvine, Ciara A. Lyons, Suneil Jain, Alan R. Hounsell, Conor K. McGarry

**Affiliations:** 1 Centre of Cancer Research and Cell Biology, Queen’s University Belfast, Belfast, BT7 1NN Northern Ireland UK; 2 Radiotherapy Physics, Northern Ireland Cancer Centre, Belfast Health and Social Care Trust, Belfast, UK; 3 Clinical Oncology, Northern Ireland Cancer Centre, Belfast Health and Social Care Trust, Belfast, UK

**Keywords:** High-risk prostate cancer, SABR, VMAT, Flattening-filter-free (FFF), Pelvic lymph nodes irradiation

## Abstract

**Background:**

The purpose of this study is to find the optimal planning settings for prostate SABR-VMAT for high-risk prostate cancer patients irradiated to prostate only (PO) or prostate and pelvic lymph nodes (PPLN).

**Methods:**

For 10 patients, plans using 6MV flattened, flattening-filter-free (FFF) 6MV (6 F) and FFF 10MV (10 F) photon beams with full and partial arc arrangements were generated and compared. The prescribed dose was 40Gy to the prostate with 25Gy to the PLN in 5 fractions. Plans were then evaluated for PTV coverage, dose fall-off, and OAR doses. The number of monitor units and the treatment delivery times were also compared. Statistical differences were evaluated using a paired sample Wilcoxon signed rank test with a significance level of 0.05%.

**Results:**

A total of 150 plans were generated for this study. Acceptable PO plans were obtained using single arcs, while two arcs were necessary for PPLN. All plans were highly conformal (CI ≥1.3 and CN ≥0.90) with no significant differences in the PTV dose coverage. 6MV plans required significantly longer treatment time and had higher dose spillage compared to FFF plans. Superior plans were obtained using 10 F 300° partial arcs for PO with the lowest rectal dose, dose spillage and the shortest treatment times. For PPLN, 6 F and 10 F plans were equivalent.

**Conclusions:**

SABR-VMAT with FFF photon beams offers a clear benefit with respect to shorter treatment delivery times and reduced dose spillage. Class solutions using a single 10 F 300° arc for PO and two 10 F or 6 F partial 300° arcs for PPLN are proposed.

**Electronic supplementary material:**

The online version of this article (doi:10.1186/s13014-016-0730-7) contains supplementary material, which is available to authorized users.

## Introduction

Stereotactic ablative body radiation therapy (SABR) has been introduced as an attractive alternative to conventional external beam radiation therapy techniques for prostate cancer patients [1–4]. In contrast with conventional techniques, SABR allows the delivery of fewer treatment fractions with higher dose per fraction (hypo-fractionation). Due to the potentially low alpha-beta ratio for the prostate (high sensitivity to fraction size), hypo-fractionation is thought to improve the therapeutic ratio for prostate radiation therapy (RT), i.e., improving tumour control rates while maintaining similar normal tissue biological effective dose compared to conventional fractionation regimens [5, 6]. Prostate SABR using volumetric modulated arc therapy (VMAT) provides highly conformal plans, with excellent tumour coverage and organs at risk (OARs) sparing [7, 8]. One major advantage for VMAT over conventional techniques is the shorter treatment times. SABR-VMAT with flattening-filter free (FFF) photon beams has been reported to be safe and effective [4, 9]. The high dose rates possible with FFF beams and the reduced leakage and scatter dose to the patient are the main reasons behind the increased use in SABR-VMAT for prostate [8–10] and prostate and pelvic lymph nodes [11].

At the Northern Ireland Cancer Centre, we are preparing for a randomized feasibility study evaluating Stereotactic PrOstate RadioTherapy in high-risk localised prostate cancer with or without elective nodal irradiation (SPORT High-Risk Trial) (http://www.hra.nhs.uk/news/research-summaries/sport-high-risk-trial/\#sthash.Bn1ByfDP.dpuf). Little is known about optimal planning techniques for SABR-VMAT for prostate cancer, especially when pelvic lymph nodes are treated electively. In preparation for the clinical introduction of SABR-VMAT through SPORT, we conducted this planning study in search of a class solution to standardize our treatment planning process. In this work, we systematically compared the use of 6MV flattened photon beam, FFF 6MV (6 F) and FFF 10MV (10 F) beams in the treatment of prostate only (PO) and prostate and pelvic lymph nodes (PPLN) in SABR-VMAT settings. Different arc arrangements were also investigated to arrive at optimal parameters for each trial arm (i.e. PO or PPLN). To the best of our knowledge, we are the first to investigate and report on the feasibility of SABR-VMAT using FFF photons with different energies and/or arc arrangements to treat prostate and prostate and pelvic lymph nodes.

## Materials and methods

Anonymized data sets from ten previously treated prostate cancer patients were selected for this study. Patients had been instructed to use enemas and follow a drinking protocol prior to imaging. CT scans with 2.5 mm slice thickness were available (from below the upper third of the femur to the top of L4).

### Contouring and planning for SABR

Contouring was aided by fusing patients’ diagnostic MRI scans with their planning CT scans in the Varian Eclipse treatment planning system (TPS) version 13.5 (Eclipse, Varian Medical Systems, Palo Alto, CA, USA). Three clinical target volumes (CTV) were defined; 1) prostate and proximal 10 mm of seminal vesicles (SV) CTV(P), 2) remaining SV CTV(SV), 3) pelvic lymph nodes CTV(LN). The planning target volumes (PTVs) were constructed to create two distinct PTVs; 1) PTV(P) which is the CTV(P) expanded with 5 mm margins in all directions except posteriorly (3 mm margin) 2) PTV(SV/LN) consisting of the CTV(SV) + CTV(LN) + 7 mm isotropic margin. Organs at risk (OARs) contoured were; bladder, rectum, sigmoid colon, bowel, femoral heads, and penile bulb. The prostatic urethra and neurovascular bundles were also contoured with reference to both the diagnostic MR and to standard anatomical references. All planning was conducted in Eclipse TPS using the Progressive Resolution Optimization (v.13.5) and Acuros XB dose calculation algorithm (v.13.5) for a Varian TrueBeam-STx Linac with a HD MLC. The dose calculation grid size used was 2.5 mm and the heterogeneity correction and jaw tracking settings were enabled. A subset of plans, were recalculated using 1.25 mm grid size to assess the effect of using a finer grid size on dose and optimization time.

### Treatment planning and planning objectives

Plans were generated utilizing 10 F and 6 F as well as 6 MV flattened photon beams. Maximum dose rates were used for each energy, 2400, 1400, and 600MU/min for 10 F, 6 F and 6MV, respectively. Several arc arrangements were investigated. Single VMAT arcs were used for PO plans [7, 8] while, after initial investigations for PPLN plans, dosimetrically acceptable plans were only achieved using two VMAT arcs.

#### Prostate only plans

Each data set was planned using three arc arrangements;

- One full 360° arc (FA).
- One partial arc 300° arc (210 → 150°; PA300).
- One partial arc 210° arc (255 → 105°; PA210) [7].

The prescribed dose was 40Gy for the CTV(P) and 36.25Gy for the PTV(P) given simultaneously in 5 fractions. Detailed planning objectives and constraints for targets and OARs are given in Table 1.

**Table 1:** Dose-volume requirements and constraints adopted for planning study

Targets	Optimal requirements	Mandatory
CTV(P) Px_1_ = 40Gy	D_98%_ ≥ 100% Px_1_	≥100% dose to ≥95 − 97%
PTV(P) Px_2_ = 36.25Gy	D_99%_ ≥95% Px_2_ V_42Gy_ ≤ 2%	≥95% dose to ≥98% volume V_42.8Gy_ ≤ 2%
PTV(SV/LN)^a^ Px_3_ = 25Gy	D_99%_ ≥95% Px_3_	≥95% dose to ≥98% volume V_107%_^b^ ≤ 2%
OARs	Optimal	Mandatory
Bladder	V_18.1Gy_ <40%	
	V_37Gy_ < 5 cc	V_37Gy_ <10 cc
Rectum	V_18.1Gy_ <50%	
	V_29Gy_ <20%	
	V_36Gy_ <1 cc	
Sigmoid	V_18.1Gy_ <50%	
	V_29Gy_ <20%	
	V_36Gy_ <1 cc	
Bowel	V_15Gy_ <78 cc	V_15Gy_ <158 cc
	V_20Gy_ <17 cc	V_20Gy_ <110 cc
	V_22.5Gy_ <14 cc	V_22.5Gy_ <28 cc
	V_25Gy_ <0.5 cc	V_25Gy_ <1 cc
Femoral heads	V_14.5Gy_ <5%	
Penile bulb	V_29.5Gy_ <50%	
Prostatic urethra	V_42Gy_ < 50%	
Neurovascular bundle	V_38Gy_ <50%	
Testes	Avoidance structure	

^a^For prostate and lymph nodes (PPLN) plans only, ^b^Evaluated for a structure [(PTV_Px_3_) - (PTV_Px_2_ + 10 mm margin)], *Px* prescription dose, *OARs* organs at risk, *V*_*XGy*_ volume receiving dose X in Gy, *D*_*X%*_ the dose received by X% of the volume

#### Prostate and pelvic lymph nodes plans

For prostate and pelvic lymph nodes planning, after initial investigations of several arc combinations, two arc arrangements were investigated further;

- Two full arcs (2FA).
- Two partial 300° arcs (210 → 150° and 150 → 210°; 2PA300).

Similar to PO plans, 40Gy was prescribed to the CTV(P) and 36.25Gy to PTV(P). Additionally, 25Gy was prescribed to the PTV(SV/LN) to be delivered simultaneously in 5 fractions. Dose objectives and constraints are also presented in Table 1.

The equivalent dose as 2Gy fractions (EQD_2Gy_) from a hypo-fractionated course for tumours and OARs could be calculated using the equation: $EQ{D}_{2 Gy}=D\left[\frac{\frac{\alpha }{\beta }+d}{\frac{\alpha }{\beta }+2}\right]$, where *D* is the total dose given at dose *d* per fraction. This hypo-fractionated dose regime corresponds to a CTV(P) EQD_2Gy_ of 108.6Gy, PTV(P) EQD_2Gy_ of 90.6Gy $\left(\frac{\alpha }{\beta }=1.5 Gy\right)$, a normal tissue late effect EQD_2Gy_ of 74.3Gy $\left(\frac{\alpha }{\beta }=3 Gy\right)$, and an acute toxicity EQD_2Gy_ of 52.1Gy $\left(\frac{\alpha }{\beta }=10 Gy\right)$.

### Plan analysis, patient specific QA and statistical analysis

For the PTVs, the near maximum dose D_2%_ and near minimum dose D_98%_ were recorded. Several dose metrics were assessed for each OAR. Additionally, dose conformity index (CI): $\frac{\mathrm{volume}\;\mathrm{of}\;95\%\;\mathrm{isodose}}{\mathrm{PTV}\;\mathrm{volume}}$ [12], conformation number (CN): $\left(\frac{\mathrm{volume}\;\mathrm{of}\;\mathrm{the}\;\mathrm{P}\mathrm{T}\mathrm{V}\;\mathrm{receiving}\;95\%\;{\mathrm{isodose}}^2}{\mathrm{PTV}\;\mathrm{volume}\times \mathrm{volume}\;\mathrm{of}\;95\%\;\mathrm{isodose}}\right)$ [12], heterogeneity index (HI): $\frac{\mathrm{dose}\;\mathrm{received}\;\mathrm{b}\mathrm{y}\;\mathrm{the}\;\mathrm{hottest}\;2\mathrm{cc}\;\mathrm{of}\;\mathrm{P}\mathrm{T}\mathrm{V}}{\mathrm{prescribed}\;\mathrm{dose}}$, were evaluated.

Population-averaged dose-volume histograms (DVHs) for the PTVs and selected OARs are presented. Medium and low-dose spillage outside the PTVs (R_50_ and R_25_) were also assessed for each plan: ${R}_x=\frac{Vol_{x\% pres}}{PTV\; volume}$, where *Vol*_*x* % *pres*_ is the tissue (body) volume receiving at least x% of the PTV prescribed dose [13]. Patient specific size parameters were also recorded and analysed for correlation with dose spillage (Additional file 1: Appendix I). The number of monitor units (MUs) and estimated treatment delivery times were also assessed.

For a subset of PO and PPLN plans, pre-treatment dose verification was conducted using OCTAVIUS-4D phantom consisting of a motorized cylindrical phantom with a ±360° angular range (PTW, Freiburg, Germany) [14]. For this investigation, a PTW OCTAVIUS 729 2*D* array was inserted into the centre of the phantom. The detector size is 0*.*5 × 0*.*5 × 0*.*5 *cm* ^*3*^ (maximum field size = 27 × 27 *cm* ^*2*^, centre to centre spacing = 10 *mm)*. Moreover, 30 plans representing high dose region, were delivered to a PTW OCTAVIUS 1000 SRS 2D array (300° partial arcs PO plans). The detector size is 2.3 mm × 2.3 mm × 0.5 mm. The detector spacing in the inner area (maximum field size = 5.5 cm × 5.5 cm) is 2.5 mm centre-to-centre and in the outer area is 5 mm centre-to-centre (maximum field size = 10 cm × 10 cm).

A *3D* gamma analysis was conducted using Verisoft software version (6.2) which creates 3*D* dose maps from the multiple 2*D* doses obtained [15]. Global gamma criteria (passing rates: 3%/3 mm ≥ 97% and 2%/2 mm ≥ 90%) were used with a 10% minimum dose threshold [16].

### Statistics

Volume data distributions were normal for some structures and non-normal for others; therefore, median values and interquartile ranges [Q1–Q3] were presented. Statistical analysis was conducted in MATLAB (v. 8.4-R2014b) using the non-parametric two-sided paired-sample Wilcoxon signed rank test as our dosimetric data was not normally distributed. As multiple comparisons were conducted, the significance level was set at *p* ≤ 0*.*005 for PO plan comparisons and *p* ≤ 0*.*01 for PPLN plans.

## Results

**Fig. 1:**
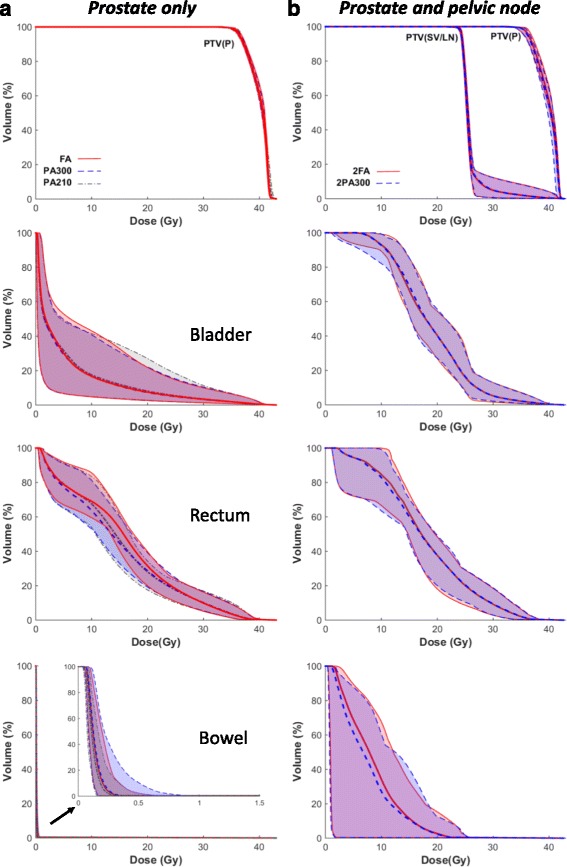
Population mean dose-volume histograms (DVHs) and standard deviation (*shaded areas*) for selected structures for 10 F plans. Left panel; prostate only (PO) plans full arc (FA), partial 300° arcs (PA300) and 210° arcs (PA210). Right panel; Prostate and pelvic nodes (PPLN) plans 2FA, 2PA300 arcs

A total of 150 plans were generated for this study. There was no significant difference in the PTV dose coverage using all energies and arc arrangements compared. CTV and PTV objectives and constraints were met in all PO and PPLN plans as shown in Tables 2 and 3. Comparing a 2.5 mm dose calculation grid size with 1.25 mm revealed only negligible differences in dose with the largest difference in PTV (P) dose of ≤1.4% in D_98%_ and D_2%_ in all cases (PO and PPLN). Five and six fold increase in calculation time for PO and PPLN plans, respectively, was observed when using a 1.25 mm grid size as opposed to 2.5 mm; therefore using a calculation grid size of 2.5 mm was favoured in this study (Additional file 1: Appendix II). Population-averaged DVHs are presented in Fig. 1 and Additional file 2: Figure S1. In all following analysis, 10 F plans were used as a reference for comparisons.

**Table 2:** Dose metrics for the PTV; median and ranges and statistical outcomes for different energies and arc arrangements for prostate only (PO) plans

Structure		FA			PA300			PA210		
Volume (cc)		10 F	6 F	6MV	10 F	6 F	6MV	10 F	6 F	6MV
	HI	1.05	1.04	1.05	1.05	1.05	1.04*	1.05	1.05	1.05
		(1.05–1.05)	(1.04–1.05)	(1.04–1.05)	(1.05–1.05)	(1.04–1.05)	(1.04–1.05)	(1.05–1.06)	(1.05–1.06)	(1.05–1.06)
	CI	1.18	1.17	1.18	1.17	1.17	1.17	1.18	1.17	1.17
		(1.17–1.19)	(1.15–1.19)	(1.15–1.19)	(1.16–1.19)	(1.16–1.19)	(1.16–1.21)	(1.17–1.21)	(1.16–1.20)	(1.16–1.20)
	CN	0.93	0.93	0.92	0.92	0.92	0.91	0.91^ɫ^	0.91	0.91
		(0.92–0.93)	(0.92–0.94)	(0.92–0.93)	(0.91–0.93)	(0.91–0.93)	(0.91–0.92)	(0.91–0.92)	(0.91–0.92)	(0.90–0.91)
	R_50_	3.03	2.94	2.97	3.12	3.16	3.11	3.31^ɫ҂^	3.43	3.35
		(2.97–3.10)	(2.90–2.99)	(2.92–3.02)	(3.10–3.24)	(3.06–3.23)	(3.02–3.19)	(3.22–3.40)	(3.38–3.48)	(3.22–3.43)
	R_25_	12.00	12.79*	12.64*	13.19	14.72*	14.06	14.24^ɫ҂^	16.3*	15.72*
		(11.42–12.52)	(12.51–13.91)	(12.04–13.10)	(12.71–13.95)	(13.90–15.37)	(13.43–14.32)	(13.65–15.57)	(15.17–17.68)	(14.75–16.53)
PTV(P)										
67.2	D_98%_ (Gy)	35.7	35.8	35.8	35.7	35.7	35.7	35.6	35.6	35.6
(54.6–92.8)		(35.6–36.0)	(35.6–36.1)	(35.6–35.9)	(35.4–36.1)	(35.6–36.1)	(35.4–35.8)	(35.5–35.7)	(35.5–35.8)	(35.3–35.75)
	D_2%_(Gy)	41.9	41.9	41.9	42.0	41.9*	41.9	42.2^҂^	42.2	42.1
		(41.9–42.0)	(41.8–41.9)	(41.8–42.0)	(41.9–42.0)	(41.8–41.9)	(41.8–41.9)	(42.0–42.3)	(41.9–42.2)	(41.9–42.3)

*Abbreviations*: *FA* full arc, *PA300* 300° partial arc, *PA210* 210° partial arc, *HI* homogeneity index, *CI* conformity index, *CN* conformation number, R_50_, R_25_ = intermediate and low dose spillage (evaluated for CTV(P) receiving 40Gy), D98% = near minimum dose, D2% = near maximum dose. * = significantly different from 10 F plans (same arc arrangement), ɫ = significantly different from 10 F (FA) plans, ҂ = significantly different from 10 F (PA300) plans

*p* ≤ 0.005 considered statistically significant (*, ɫ, ҂)

**Table 3:** Dose metrics for PTVs; median and ranges and statistical outcomes for different energies and arc arrangements for prostate and pelvic nodes (PPLN) plans

Structure		2FA			2PA300		
Volume (cc)		10 F	6 F	6MV	10 F	6 F	6MV
	HI	1.05	1.05	1.05	1.05	1.05	1.05
		(1.05–1.05)	(1.05–1.05)	(1.05–1.05)	(1.05–1.05)	(1.05–1.05)	(1.05–1.05)
	CI	1.21	1.21	1.22	1.23 ^ɫ^	1.23	1.22
		(1.20–1.22)	(1.18–1.22)	(1.20–1.22)	(1.21–1.24)	(1.21–1.24)	(1.21–1.24)
	CN	0.91	0.91	0.91	0.91	0.91	0.91
		(0.89–0.92)	(0.90–0.92)	(0.91–0.92)	(0.88–0.92)	(0.89–0.92)	(0.89–0.92)
	R_50_	4.21	4.43*	4.50*	4.31	4.63*	4.64*
		(4.06–4.39)	(4.31–4.60)	(4.42–4.67)	(4.17–4.43)	(4.48–4.81)	(4.57–4.79)
	R_25_	8.65	9.02*	9.19*	8.80	9.09	9.19*
		(8.32–9.07)	(8.63–9.63)	(8.69–9.72)	(8.48–9.24)	(8.70–9.43)	(8.83–9.81)
PTV(P)							
67.2	D_98%_ (Gy)	35.7	35.8	35.9	35.7	35.8	35.8
(54.6–92.8)		(35.3–36.1)	(35.4–36.0)	(35.5–36.1)	(35.3–36.0)	(35.3–35.9)	(35.4–35.9)
	D_2%_ (Gy)	42.0	42.0	42.0	42.0	42.0	42.1
		(42.0–42.1)	(42.0–42.1)	(42.0–42.1)	(42.0–42.1)	(42.0–42.1)	(42.0–42.1)
PTV(SV/LN)							
796.2	D_98%_(Gy)	24.2	24.2	24.2	24.1	24.0	24.1
(746.4 − 855.9)		(24.1–24.3)	(24.1–24.2)	(24.2–24.3)	(24.0–24.2)	(23.9–24.0)	(24.1–24.3)
	D_2%_ (Gy)^¥^	26.6	26.6	24.7	26.6	26.7	26.7
		(26.6–26.7)	(26.5–26.7)	(26.5–26.7)	(26.6–26.8)	(26.6–26.8)	(26.6–26.8)

*Abbreviations*: *FA* full arc, *PA300* 300° partial arc, *HI* homogeneity index, *CI* conformity index, *CN* conformation number, *R*_*50*_ *, R*_*25*_ intermediate and low dose spillage (evaluated for PTV(SV/LN) receiving 25Gy), D98% = near minimum dose, D2% = near maximum dose. * = significantly different from 10 F plans (same arc arrangement), ɫ = significantly different from 10 F (FA) plans. ¥ assessed for a structure [(PTV(SV/LN)) – (PTV(P) = 10 mm]

*p* ≤ 0.01 considered statistically significant (*, ɫ)

### Prostate only plans

Dosimetrically, all evaluated plans were highly conformal CI = 1.17 − 1.18 and CN ≥ 0*.*91, Table 2. For FA plans, a slight, but significant, increase in low-dose (*R*_25_) spillage outside the PTV was observed in 6 F and 6MV flattened beams plans compared to 10 F plans (*p* = 0*.*0020 in both cases). The same trend was observed in partial arc plans with significantly lower (*R*_25_) values for plans delivered with 10 F photon beams. Compared to FA plans, 10 F plans with partial 300° arcs also resulted in a slight increase in the *R*_50_ and *R*_25_; (FA vs. PA300: 3.0 vs 3.1 (*p* = 0*.*0195) and 12.0 vs 13.2 (*p* = 0*.*0371), respectively). PA210 plans had the highest intermediate- and low-dose spillage outside PTV (FA vs. PA210; *R*_50_ 3.03 vs. 3.31 (*p* = 0*.*0039) and *R*_25_ 12.00 vs. 14.24 (*p* = 0*.*0039)). Table 4 shows OAR dose metrics resulting from using different energies and beam arrangements.

**Table 4:** Dose metrics for OARs; median and ranges and statistical outcomes for different energies and arc arrangements for prostate only (PO) plans

Structure		FA			PA300			PA210		
Volume (cc)		10 F	6 F	6MV	10 F	6 F	6MV	10 F	6 F	6MV
Bladder	Mean (Gy)	4.1	4.3	4.3*	4.1	4.2	4.0	4.4^҂^	4.5*	4.6*
448.2		(2.6–8.7)	(2.7–8.8)	(2.7–8.8)	(2.4–8.7)	(2.6–8.7)	(2.6–8.6)	(2.5–9.0)	(2.6–9.2)	(2.6–9.2)
(234.3 − 566.0)	D_2cc_(Gy)	40.0	40.1	40.1	40.3	40.2	39.8	40.0	39.9	39.9
		(39.6–40.6)	(39.4–40.5)	(39.5–40.6)	(39.4–40.6)	(39.6–40.6)	(39.5–40.2)	(39.3–40.2)	(39.4–40.3)	(39.5–40.4)
	D_95%_ (Gy)	0.2	0.4*	0.3*	0.2	0.3*	0.3	0.2	0.3*	0.3*
		(0.1–0.6)	(0.2–0.8)	(0.2–0.8)	(0.1–0.6)	(0.2–0.8)	(0.2–0.8)	(0.1–0.6)	(0.2–0.9)	(0.2–0.8)
	D_50%_ (Gy)	0.8	1.1	1.1	0.8	1.1	0.9	0.8	1.1	1.1
		(0.6–2.6)	(0.8–2.6)	(0.8–2.8)	(0.5–2.5)	(0.8–2.4)	(0.7–2.5)	(0.5–2.5)	(0.8–2.4)	(0.7–2.5)
	V_18.1Gy_ (%)	8.4	8.1	8.4	8.3	8.5	7.8	9.8 ^ɫ҂^	9.7	10.0
		(4.5–20.0)	(4.4–19.9)	(4.3–19.7)	(4.2–20.3)	(4.2–20.8)	(4.2–20.3)	(4.7–22.6)	(4.6–23.4)	(4.7–23.1)
	V_29Gy_ (%)	3.6	3.4	3.5*	3.7	3.6	3.0	4.0^҂^	4.0	4.0
		(2.6–8.0)	(2.5–7.8)	(2.5–7.8)	(2.4–7.8)	(2.4–7.9)	(2.5–7.9)	(2.5–9.0)	(2.5–9.0)	(2.5–9.1)
	V_37Gy_ (cc)	7.4	7.5	7.5	7.5	7.4	6.6	7.1	7.1	7.2
		(5.6–7.7)	(5.5–7.9)	(5.5–7.8)	(5.6–7.8)	(5.6–7.9)	(5.0–7.7)	(5.7–7.8)	(5.6–7.5)	(5.3–7.7)
Rectum	Mean(Gy)	16.0	16.0	15.8	14.4 ^ɫ^	14.7	14.3	15.1 ^ɫ^	14.8	14.7
57.9		(14.1–16.9)	(14.2–16.8)	(14.1–17.0)	(13.2–16.2)	(13.1–16.4)	(12.6–15.7)	(13.2–16.7)	(13.3–15.9)	(13.3–16.4)
(53.8–72.2)	D_2cc_ (Gy)	35.6	35.7	35.7	35.6	35.6	35.0	35.8	35.9	35.8
		(32.1–37.5)	(32.0–37.3)	(31.7–37.6)	(32.6–37.6)	(32.6–37.5)	(31.5–37.1)	(32.6–37.6)	(33.3–37.5)	(32.7–37.6)
	D_95%_ (Gy)	1.2	1.4	1.4*	1.1	1.4	1.4	1.2	1.4	1.4*
		(1.0–1.9)	(1.2–2.0)	(1.3–2.2)	(1.0–1.9)	(1.2–2.0)	(1.3–1.7)	(1.0–2.1)	(1.2–2.0)	(1.2–2.1)
	D_50%_ (Gy)	15.7	15.7	15.3	14.2 ^ɫ^	13.9	13.4	14.3 ^ɫ^	13.4	13.7
		(14.7–17.0)	(14.5–17.0)	(14.8–16.8)	(11.4–14.9)	(11.7–15.6)	(11.3–15.0)	(12.7–15.7)	(12.5–14.6)	(12.2–15.7)
	V_18.1Gy_ (%)	38.6	38.7	38.1	34.0	34.2	33.8	34.6 ^ɫ^	30.6	31.1
		(34.7–43.5)	(34.8–43.1)	(33.6–43.1)	(30.1–39.1)	(29.8–38.8)	(26.3–37.5)	(29.1–39.3)	(27.6–37.7)	(28.3–39.7)
	V_29Gy_ (%)	12.1	11.8	11.7	11.0	11.5	10.8	11.2	11.2	11.4
		(6.6–15.4)	(6.3–15.3)	(6.1–15.1)	(7.2–16.2)	(7.1–15.0)	(6.2–14.2)	(6.6–15.6)	(7.4–15.9)	(6.9–15.8)
	V_36Gy_ (cc)	1.9	1.9	1.9	1.9	1.9	1.2	2.0	2.0	2.0
		(0.6–2.7)	(0.7–2.7)	(0.6–2.7)	(0.6–2.7)	(0.8–2.7)	(0.6–2.4)	(0.9–2.8)	(0.9–2.8)	(0.8–2.8)
	V_38Gy_ (cc)	0.7	0.7	0.7	0.6	0.6	0.4	0.7	0.8	0.7
		(0.1–1.3)	(0.1–1.2)	(0.1–1.3)	(0.1–1.3)	(0.1–1.3)	(0.1–1.0)	(0.2–1.3)	(0.2–1.3)	(0.2–1.4)
Sigmoid	Mean (Gy)	0.6	0.8*	0.8*	0.6	0.8*	0.8	0.6	0.8*	0.8*
106.1 (89.5–156.8)		(0.3–0.9)	(0.4–1.0)	(0.4–1.0)	(0.3–0.9)	(0.4–1.0)	(0.4–1.0)	(0.3–0.9)	(0.4–1.0)	(0.4–1.0)
Bowel	Mean (Gy)	0.1	0.2*	0.2*	0.2	0.2*	0.2	0.1	0.2*	0.2*
255.2 (95.1–376.9)		(0.1–0.2)	(0.2–0.3)	(0.1–0.2)	(0.2–0.2)	(0.1–0.2)	(0.1–0.2)	(0.1–0.2)	(0.2–0.2)	(0.1–0.2)
Rt Fem	Mean (Gy)	5.7	6.1	6.3	7.1	7.3	6.3	7.8 ^ɫ҂^	8.0	8.0
52.6 (51.1–57.5)		(4.8–6.6)	(4.7–7.9)	(4.7–8.1)	(5.3–8.1)	(5.3–8.2)	(4.8–8.0)	(7.0–8.6)	(6.8–9.1)	(6.2–8.7)
Lt Fem	Mean (Gy)	6.4	6.5	6.1	6.6	7.1	6.5	7.4	7.8	7.4
55.1 (50.5–57.5)		(4.5–7.7)	(4.8–7.4)	(4.6–8.2)	(5.0–7.6)	(5.8–7.9)	(5.1–8.6)	(5.5–8.5)	(5.6–9.2)	(5.8–9.0)
Urethra	V_42Gy_ (%)	0.1	0.1	0.0	3.1	0.0	0.0	5.6	1.3	3.0
0.4 (0.3–0.6)		(0.0–5.5)	(0.0–0.4)	(0.0–4.3)	(0.0–6.8)	(0.0–5.0)	(0.0–3.7)	(0.0–40.6)	(0.0–9.0)	(0–21.0)
NV Bundle	V_38Gy_ (%)	32.2	33.7	31.5	27.3	33.6	27.0	24.9	40.2	40.0
0.8 (0.3–2.4)		(15.8–42.7)	(19.5–41.4)	(18.2–47.0)	(14.8–39.7)	(19.3–41.2)	(18.7–38.8)	(9.4–45.2)	(39.0–40.7)	(39.0–41.0)

*Abbreviations*: *FA* full arc, *PA300* 300° partial arc, *PA210* 210° partial arc, *D*_*2cc*_ the dose received by the hottest 2 cc (near maximum dose), *D*_*X%*_ the dose received by X% of the volume, *V*_*XGy*_ the volume receiving dose XGy. *Rt* right, *Lt* left, *Fem* femoral head, *NV* Neurovascular, ^*^ = significantly different from 10 F plans (same arc arrangement), ^ɫ^ = significantly different from 10 F (FA) plans, ^҂^ = significantly different from 10 F (PA300) plans

*p* ≤ 0.005 considered statistically significant (*, ɫ, ҂)

Overall, only small dosimetric differences exist between the different plans. For PO plans, 10 F beams with PA300 arcs were superior compared to other energies and beam arrangements leading to significantly lower doses to the rectum (mean dose and *D*_50%_ for FA 10 F vs. 10 F PA300: 16Gy vs. 14.4Gy (*p* = 0*.*0039) and 15.7Gy vs. 14.2Gy (*p* = 0*.*002), respectively).

### Prostate and pelvic lymph nodes plans

Only minor differences were observed between the different PPLN plans in terms of PTV coverage, dose spillage and doses to OAR as seen in Tables 3 and 5. It was not always possible to meet all OARs dose-volume constraints adopted for PO plans, therefore minor relaxation of uppermost bladder and rectum constraints were permitted. Interactive attempts were made to keep the OARs doses as low as possible without jeopardising PTVs coverage (Fig. 1).

**Table 5:** Dose metrics for OARs; median and ranges and statistical outcomes for different energies and arc arrangements for prostate and pelvic nodes (PPLN) plans

Structure		2FA			2PA300		
Volume (cc)	metric	10 F	6 F	6MV	10 F	6 F	6MV
Bladder	Mean (Gy)	19.1	19.1	19.3	19.3	19.5	19.3
448.2		(17.0–20.3)	(17.2–20.3)	(17.0–20.4)	(17.4–20.4)	(18.1–20.3)	(17.6–20.2)
(234.3 − 566.0)	D_2cc_ (Gy)	39.4	39.4	39.5	39.5	39.3	39.3
		(38.8–40.0)	(38.9–40.0)	(38.9–40.1)	(39.1–40.0)	(39.0–40.0)	(39.1–40.0)
	D_95%_ (Gy)	9.5	10.4	10.6	10.1	10.8	10.8
		(8.3–10.8)	(8.7–11.1)	(8.4–11.7)	(7.5–11.6)	(8.1–11.5)	(8.4–11.7)
	D_50%_ (Gy)	18.5	18.3	18.4	18	18.1	17.9
		(15.7–19.6)	(15.9–19.5)	(15.8–19.4)	(16.5–19.6)	(17.0–19.8)	(16.5–19.2)
	V_18.1Gy_ (%)	51.3	50.2	50.6	49.4	49.8	48.9
		(38.3–57.4)	(37.3–57.7)	(37.2–57.0)	(42.8–61.7)	(44.3–60.5)	(42.2–57.7)
	V_37Gy_ (cc)	7.2	6.9	6.8	7.1	6.9	6.5
		(4.9–7.5)	(5.0–7.5)	(4.6–7.6)	(5.8–7.5)	(5.1–7.3)	(5.1–7.5)
Rectum	Mean (Gy)	18.6	18.7	18.7	18.4	18.4	18.5
57.9		(16.9–19.7)	(16.7–19.4)	(16.9–19.7)	(16.7–19.7)	(16.7–19.8)	(16.7–19.8)
(53.8–72.2)	D_2cc_ (Gy)	34.8	34.7	34.7	34.8	34.9	34.8
		(31.2–36.0)	(30.7–36.0)	(30.6–35.9)	(31.1–36.0)	(31.3–36.1)	(30.8–36.0)
	D_95%_ (Gy)	3.7	3.8	4.1*	4.1	4.0	4.5
		(1.7–7.3)	(2.2–6.9)	(2.5–7.5)	(1.8–7.7)	(2.3–7.3)	(2.6–7.6)
	D_50%_ (Gy)	17.2	17.2	17.1	17.4	17.1	17.3
		(16.0–18.6)	(16.0–18.4)	(16.1–18.3)	(15.9–18.2)	(15.9–18.2)	(16.0–17.9)
	V_18.1Gy_ (cc)	45.4	45.2	45.3	46.7	45.5	45.9
		(40.1–52.0)	(39.9–51.2)	(41.1–50.6)	(40.2–49.8)	(39.9–50.1)	(41.1–49.0)
	V_29Gy_ (%)	11.6	12.2	11.9	11.1	11.2	11.3
		(6.0–17.8)	(5.4–16.4)	(5.3–17.3)	(6.1–18.4)	(6.1–18.4)	(6.0–18.9)
	V_36Gy_ (cc)	1.0	1.1	1.0	1.1^ɫ^	1.1	1.1
		(0.3–2.0)	(0.2–2.1)	(0.2–2.0)	(0.5–2.2)	(0.3–2.1)	(0.2–2.1)
	V_38Gy_ (cc)	0.1	0.1	0.1	0.1	0.1	0.1
		(0.0–0.3)	(0.0–0.3)	(0.0–0.2)	(0.0–0.3)	(0.0–0.3)	(0.0–0.3)
Sigmoid	Mean (Gy)	18.7	18.7	18.7	19.1	19.2	18.9
106.1		(12.5–20.4)	(12.6–20.3)	(12.8–20.1)	(12.5–20.4)	(12.7–20.8)	(13.1–20.5)
(89.5–156.8)	V_18.1Gy_ (cc)	58.5	57.5	56.8	59.3	59.7	59.1
		(36.3–67.1)	(33.6–65.7)	(35.3–64.4)	(36.3–67.1)	(36.1–69.7)	(37.8–67.2)
Bowel	Mean (Gy)	9.3	9.5	9.1	8.4	8.4	8.2
255.2		(6.1–11.5)	(6.1–11.7)	(6.3–11.7)	(5.9–11.5)	(6.2–11.7)	(6.4–11.8)
(95.1–376.9)	V_15Gy_ (cc)	38.8	40.6	35.9	46.9	42.7	42.2
		(5.6–80.1)	(5.4–82.5)	(3.8–82.0)	(7.2–70.7)	(8.7–80.6)	(7.1–74.8)
	V_20Gy_ (cc)	10.0	11.0	9.4	11.9	10.9	11.9
		(2.4–29.7)	(2.3–30.5)	(0.9–29.6)	(2.4–31.4)	(2.9–33.9)	(2.6–33.4)
	V_22.5Gy_ (cc)	5.5	5.5	5.3	5.8	5.7	5.4
		(1.5–16.1)	(1.4–16.1)	(0.4–16.1)	(1.3–17.9)	(1.7–17.3)	(1.5–16.7)
	V_25Gy_ (cc)	1.3	1.5	1.4	1.7	1.3	1.8
		(0.5–2.4)	(0.4–2.5)	(0.1–2.5)	(0.3–3.6)	(0.6–3.2)	(0.5–2.9)
Rt Fem	Mean (Gy)	10.4	11.1	11.1	10.4	11.1*	10.2
52.6 (51.1–57.5)		(10.2–11.2)	(10.5–11.3)	(10.7–11.5)	(9.2–11.1)	(9.4–11.3)	(9.3–11.3)
Lt Fem	Mean (Gy)	10.5	10.8	10.9	9.9	10.4*	10.3*
55.1 (50.5–57.5)		(9.7–11.1)	(10.1–11.4)	(10.6–11.5)	(9.1–11.0)	(9.2–11.9)	(9.5–11.4)
Penile B	V_29.5Gy_ (cc)	3.2	3.1	3.5*	3.3	3.1	3.4
3.4 (2.3–4.4)		(1.7–8.6)	(2.1–8.5)	(2.4–8.8)	(1.7–8.4)	(2.1–8.0)	(2.4–8.5)
Urethra	V_42Gy_ (%)	3.6	0.5	0.7	1.4	5.7	0.6
0.4 (0.3–0.6)		(0.0–14.6)	(0.0–10.4)	(0.0–7.4)	(0.0–16.7)	(0.0–13.2)	(0.0–27.0)
NV Bundle	V_38Gy_ (%)	35.8	34.7	33.7	25.5	28.0	26.5
0.8 (0.3–2.4)		(7.7–45.8)	(4.9–45.5)	(9.3–42.2)	(9.8–42.7)	(12.4–41.8)	(11.9–42.1)

*Abbreviations*: *FA* full arc, *2PA300* 300° partial arcs, *D*_*2cc*_ the dose received by the hottest 2 cc (near maximum dose), *D*_*X%*_ the dose received by X% of the volume, V_XGy_ = the volume receiving dose XGy. *Rt* right, *Lt* left, *Fem* femoral head, *Penile B* penile bulb, *NV* Neurovascular, ^*^ = significantly different from 10 F plans (same arc arrangement), ^ɫ^ = significantly different from 10 F (FA) plans

*p* ≤ 0.01 considered statistically significant (*, ɫ)

Comparing PO plans with PPLN plans, there was a significant increase in the dose to OARs (10 F mean doses, PO PA300 vs. PPLN 2PA300; bladder (4.1(2.4 − 8.7) Gy vs. 19.3(17.4 − 20.4) Gy), rectum (14.4(13.2 − 16.2) Gy vs. 18.4(16.7 − 19.7) Gy), Bowel (0.2(0.2 − 0.2) Gy vs. 8.4(5.9 − 11.5) Gy).

### MUs and estimated delivery times

**Fig. 2:**
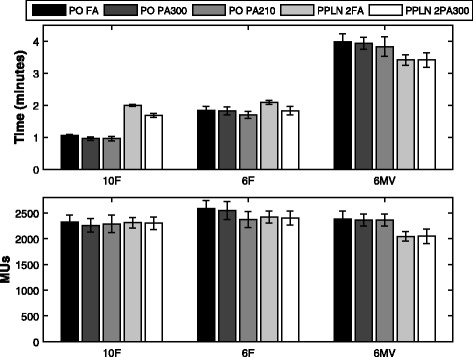
Population mean and standard deviation (*error bars*) for the estimated delivery times (*upper panel*) and monitor units (*lower panel*) for all plans investigated

Highly significant differences in MUs and delivery times emerged between plans when using different energies as shown in Fig. 2. Superior plans were obtained using 10 F beams in terms of treatment time for both PO and PPLN plans. This reduction was more pronounced in PO plans where on average 73% (*p* = 0*.*002) and 43% (*p* = 0*.*002) reduction in treatment time was obtained when using 10 F beams compared to 6MV and 6 F, respectively. Using PA300 arcs, a further small but statistically significant reduction in treatment time was obtained (PA300 10 F vs. FA10F: 57.4 ± 4.2 vs. 63.3 ± 2.3 seconds(s), *p* = 0*.*002). A slight reduction in MUs was also observed in PA300 plans (10 F PA300 vs. 10 F FA: 2195 ± 87 vs. 2362 ± 27 MUs) but was found insignificant.

As shown in Fig. 2, the mean MUs and delivery times for 2FA PPLN plans were; 2312 ± 102 and 120.0 ± 1.9 s for 10 F beams, 2423 ± 115 and 125.3 ± 3.8 s for 6 F beams, 2046 ± 97 s and 204.6 ± 9.7 s for 6MV beams, respectively. For PPLN plans, 6MV plans had the lowest number of MUs compared to FFF beams; this did not however, translate into shorter treatment times due to the limitation of the maximum dose rate associated with 6MV beams (600MU/min). In terms of treatment time, PPLN 10 F and 6 F plans were equivalent and both more efficient than plans delivered using 6MV beams (10 F vs 6 F vs 6MV: 120 vs. 125 vs. 205 s, respectively). Compared to 2FA plans, a further 15.6% and 12.7% reduction in treatment time was obtained when using partial 10 F and 6 F arcs (2PA300), respectively.

### Patient specific QA

**Fig. 3:**
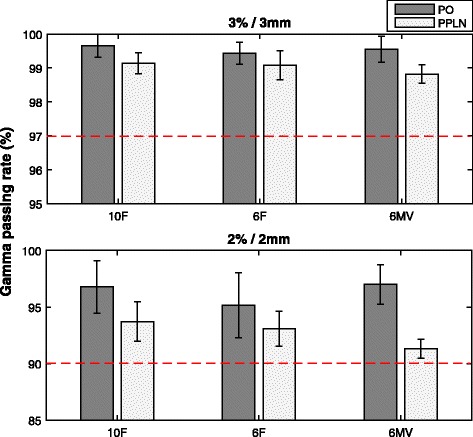
Results of pre-treatment patient specific QA gamma analysis (PTW OCTAVIUS 729 2*D* array). Mean gamma passing rate and standard deviation for 3%/3 mm (*upper panel*) and 2%/2 mm (*lower panel*) global gamma analysis 300° partial arcs plans for PO and PPLN delivered with different photon energies

Results of gamma analysis for a subset of plans are presented Fig. 3, (60 plans; PA300 plans for PO and PPLN for all energies investigated, also see Additional file 1: Appendix III for results obtained using the OCTAVIUS 1000 SRS array (30 PO plans)). Gamma passing rates were slightly lower in PPLN compared to PO plans; however, all plans were dosimetrically accepted using our clinical acceptance thresholds as shown in Fig. 3.

## Discussion

In this study, we systematically evaluated and compared the use of 10 F, 6 F, and 6MV photon beams for SABR-VMAT PO and PPLN planning. Our results demonstrate that it is feasible to treat the prostate with/without pelvic nodes using flattened and un-flattened photon beams. Optimal plans were obtained using FFF beams in terms of the low-dose bath outside the PTVs and significantly shorter treatment times for both PO and PPLN plans. For PO, 10 F plans using partial 300° arcs were superior to other energies and beam arrangements, leading to significantly lower rectal doses with the shortest treatment time. This came at the expense of a slight increase in the *R*_50_ and *R*_25_ compared to FA plans. No further reduction in treatment time or OAR doses was observed when using 210° arcs, and a further significant increase in dose spillage outside the PTV was present. In PPLN plans, only minor differences in the low-dose region were observed between 10 F and 6 F plans, although both were superior to 6MV plans for all arc arrangements. Once more, 2PA300 resulted in dosimetrically equivalent plans and shorter treatment time compared to 2FA plans in both 10 F and 6 F plans.

In a recent planning study, VMAT plans for patients with different body habitus (*n* = 40) were generated using 6MV and 10MV flattened beams for PPLN (54Gy in 30 fractions) [13]. The authors concluded that 10MV plans were better, providing faster dose falloff, and this improvement increased linearly with increasing patient size. In this current study, only minor differences (clinically insignificant) were observed in plans quality using 10 F and 6 F photons for PPLN (Tables 3 and 5 and Additional file 1: Appendix I for further analysis).

Several studies have shown that using a single VMAT arc in PO plans is favoured over two arcs, being dosimetrically equivalent and requiring shorter treatment times [7, 8]. Shorter delivery times minimize the risk of intra-fraction motion which is highly relevant, especially in the SABR settings. A class solution with a single partial arc of 210° for prostate only VMAT-SABR was proposed by Murray et al. (6MV, 42.7Gy in 7 fractions) [7]. Compared to FA plans, 210° arcs plans had reduced rectal dose, MUs and estimated treatment times [7]. The different optimal arc of PA300 proposed in the present study could be due to the different planning system/Linacs used (Elekta vs Varian). In an investigation of the use of FFF single arc vs. dual arcs VMAT techniques for low-, intermediate- and high-risk prostate cancer, Fortin et al. also favoured the use of a single arc (FA) in all risk groups planned (10 F, 36.25Gy in 5 fractions) [8]. However, their definition of CTV for high-risk patients was the prostate + lower 2 cm SV and did not include PLN, once more making their plans equivalent to our presented PO plans. Despite the differences between our study and the available literature [7, 8], good agreements were found in target coverage, conformity and OAR doses.

As reported in the literature [10] and confirmed in this study, the major advantage of using un-flattened photon beams in SABR-VMAT over flattened beams is the considerable reduction in treatment time. Using our proposed class solutions, a delivery time of less than a minute was required for PO plans, while PPLN plans were delivered in less than two minutes. Similar treatment times were reported by Fortin et al. [8] for PO plans using 10 F single and dual VMAT arcs.

In a clinical investigation of 5 fractions SABR (40Gy to prostate and 25Gy to PLN) with brief androgen suppression (12 months) for high-risk prostate cancer, Bauman et al. reported higher than anticipated late rectal toxicity that led to trial termination after recruiting 16 patients [11]. High rates of acute (26%) and late (60%) grade 2 toxicity were observed. Four patients (26%) experienced late grade 3 GI toxicity and one patient (7%) developed a grade 4 GI toxicity. In their protocol, 25Gy was prescribed to (lymph node + 5 mm isotropic margin) and 40Gy to the (prostate + 1 cm proximal SV + 5 mm). The authors acknowledged the limitations of their study and suggested that several confounding factors; specific frail elderly patient group, contouring on CT scans alone, large high-dose PTV, relaxed OARs constraints, lack of image guidance and manual registration with no fiducial markers were among the candidate factors for such high toxicities. In their letter to editors, Kishan et al. argued that the high toxicity reported by Bauman et al. [11] originated neither from the prostate being prescribed 40Gy nor the inclusion of the PLN, as their preliminary results in a similar study with a different design, suggests that both approaches were well tolerated by their patient group [17]. In a recent study (the largest of its kind) of predictive parameters for rectal bleeding grade 2 or higher in prostate SABR, multivariate modelling revealed that the rectal volume receiving ≥38Gy (*EQD*_2*Gy*_ = 80.6Gy for $\frac{\alpha }{\beta }=3$) is a strong predictor for high grade haematochezia [18]. In our study, despite the increased rectal doses in PPLN compared to PO plans, only negligible rectal volumes received 38Gy (0.0 − 0.3 cc), which is well below the 2 cc recommended threshold [18].

Emerging results from several clinical trials of dose escalation with SABR (35Gy − 50Gy in 5 fractions) show excellent biochemical control and low to moderate late toxicities [19–22]. This is also confirmed by the early results from the ongoing SATURN SABR trial including lymph nodes for high-risk prostate [23]. In the SPORT trial design, after initial CT scanning, patients will have three gold seed fiducial markers for image-guidance. From our experience and also as noted from the literature, the biggest challenge in SABR is to meet bladder and rectal dose constraints. For each patient, a polyethylene glycol hydrogel spacer will be inserted under trans-rectal ultrasound guidance which will potentially lead to further rectal dose reduction [24, 25]. Simulation CT and MRI scans will then be acquired and fused for contouring.

As discussed by Marino et al [26], treatment plan quality could also be influenced by several other factors related to setting priorities to create the right balance between PTV coverage and OAR sparing. Personal knowledge of treatment planning systems and the experience of the planner are among the factors affecting the quality of treatment plans. Using a class solution for planning will provide a simple method to address these problems and optimize treatment planning for SABR prostate. Further improvements in creating more consistent high quality plans with minimal work load could be anticipated when using advanced methods, e.g. knowledge-based (semi-automated) planning [27], multi-criteria optimization [28], plan quality assurance models and/or systems capable of fully automated treatment planning [29].

To the best of our knowledge, this is the first study to investigate an optimal SABR-VMAT planning class solution for prostate and pelvic lymph nodes. The efficacy of several arc arrangements and different photon beam energies was assessed and class solutions are presented. To achieve the anticipated theoretical benefits of SABR without introducing unnecessary toxicity risk, strict protocols should be set and followed to control and minimize treatment uncertainty and to ensure the delivery of the dose levels accepted at planning. Potentially lower rectal doses could be achieved by using anatomy modifiers; however, it is more difficult to reduce bladder doses further, especially when the lymph nodes are electively irradiated. Therefore, short and long term follow-up is warranted to enable a full assessment of each treatment arm.”

## Conclusions

Treatment plans using FFF photon beams required shorter delivery times and demonstrated reduced dose spillage outside the PTV compared to plans obtained using flattened beams. A class solution employing a single 300° partial arc with 10 F photons is optimal for SABR-VMAT for prostate only. Two 300° partial arcs of either 6 F or 10 F photon beams are equally optimal to deliver acceptable plans for prostate and pelvic lymph node SABR-VMAT. Caution must be applied when planning exceptionally large patients as the proposed class solution may not be optimal.

## Additional files

Additional file 1: A study of plan quality robustness with varying patient size: Table S1-Table S3: Figure S2- Figure S5. (DOCX 29 kb)
Additional file 2: Figure S1. Population mean dose-volume histograms (DVHs) and standard deviation (shaded areas) for selected structures for full arc (FA) plans using different energies. Left panel: prostate only (PO) single FA plans. Right panel: prostate and pelvic nodes (PPLN) dual full arc (2FA) plans. (PDF 350 kb)

## Abbreviations

CI: Conformity index
CN: Conformation number
CTV: Clinical target volume
DVH: Dose volume histogram
EQD_2Gy_: Equivalent dose as 2Gy fractions
FA: Full arc
FFF: Flattening-filter-free
HI: Heterogeneity index
OAR: Organ at risk
PA: Partial arc
PLN: Pelvic lymph nodes
PO: Prostate only
PPLN: Prostate and pelvic lymph node
PTV: Planning target volume
QA: Quality assurance
SABR: Stereotactic ablative body radiation therapy
SPORT: Stereotactic prostate RT in high-risk localised prostate cancer with or without elective nodal irradiation
SV: Seminal vesicles
TPS: Treatment planning system
VMAT: Volumetric modulated arc therapy
