# Supplementary material for: Class solutions for SABR-VMAT for high-risk prostate cancer with and without elective nodal irradiation
Source: Radiat Oncol. 2016 Nov 24;11:155. doi: 10.1186/s13014-016-0730-7 (PMC5121961; doi:10.1186/s13014-016-0730-7)
Supplement: Additional file 1: — A study of plan quality robustness with varying patient size: Table S1-Table S3: Figure S2- Figure S5. (DOCX 29 kb) [file 13014_2016_730_MOESM1_ESM.docx]

**Additional document, Appendix I:**

**Plan quality robustness with varying patient size**

In this analysis, changes in the dose spillage outside PTVs with varying body size were investigated. Simple regression analysis was conducted to quantify these changes [1]. Body size effects on plans produced using different energies and arc arrangements were also tested.

**Materials and methods:**

### Patient specific size parameters

Three metrics were recorded for each patient; the lateral thickness of the patient (LR), the anterior-posterior thickness (AP) as measured from CT at the centre of the CTV(P) and the target to body volume ratio (TBV) [1]. The target considered is the PTV(P), and the body contour (*Vol_EXT_*) was restricted to the axial slices where the PTV(P) is present $\left( TBV=\frac{PTV(P)}{\mathrm{Vol}_{\mathrm{EXT}}} \right)$ [1]. Employing the Shapiro-Wilk test of normality in SPSS (version 22), it was revealed that our investigated parameters were mixed normally and non-normally distributed; therefore, median values and interquartile ranges [Q1-Q3] are presented. Correlation testing and regression analysis was conducted using MATLAB (version 8.4.0.150421 (R2014b)).

**Results and discussion:**

Supplementary table 1 shows a summary of patients’ characteristics and body habitus for the patients in this study. Population mean PTV(P) volume (median(range)) was 67.2 (54.6 − 92.8)cc, and PTV(SV/LN) volume was 796.2(746.4−855.9)cc. Population mean patient thickness was; AP= 21.2(19.9 −23.2)cm and LR=35.4(34.6 −37.6) cm, and the mean TBV ratio =0.016(0.016−0.019).

Correlation analysis was performed to test correlations between low- and intermediate-dose spillage and the different measured size parameters (LR, AP, and TBV). No statistically significant correlation was found for dose spillage as a function of TBV. Moreover, employing Pearson correlation coefficients, a strong positive linear correlation was found between AP and LR patient thickness (ρ = 0.85, p=0.002). Therefore, only the regression analysis with AP thickness are presented in supplementary Figures 2−4. In the following sections, ‘patient size’ is used as a proxy for AP thickness of the patient.

No significant correlation was found for high-dose spillage as a function of patient size in PO plans. Comparing PO plans with 6F and 10F (supplementary Fig. 2), R_25_ increased linearly with increasing body size as could be seen in the divergence of the regression lines with increasing size. 10F plans seems to be more robust against patient size in both FA and PA300 plans, as reflected by the smaller slopes for 10F regression lines compared to 6F and 6MV (supplementary Fig. 2(a) and 2(b)).

For PPLN plans with larger PTVs, once more, 10F plans had the lowest (intermediate- and high-) dose spillage. Overall, a small increase in R_50_ and R_25_ was observed in 6F plans compared to 10F plans. For 6F 2FA plans, the percentage difference in R_50_ compared to 10F plans was not patient size dependent, Supplementary Fig. 3(c). However, in partial arcs plans a marginally significant increase in plans R_50_ as a function of patient size was observed for 6F plans (supplementary Fig. 3(d)). This indicates that using 10F may be beneficial for large patients with reduced R_50_ compared to 6F. From supplementary Fig. 4(b), 4(d), it could be seen that for 2PA300 plans, the nearly parallel regression lines show that R_25_ increased with increasing body size reflecting the robustness of both 10F and 6F plans against patient size for the range of patient sizes investigated in this study. Comparing PO and PPLN, the percentage difference in R_25_ between 6F plans and 10F plans seems to be more pronounced in PO plans.

To summarize, for PO planning the use of 10F photon beams is advised as there is a clear benefit in terms of low-dose spillage especially for larger patients. In PPLN planning using our class solution (2PA300), both 10F and 6F are robust against varying body size. Exceptionally large patients should be planned with care as these conclusions might not hold.

**Additional document, Appendix II:**

**Comparison of 2.5mm and 1.25 mm dose calculation grid size.**

Comparison of 2.5mm grid size with 1.25mm revealed only negligible differences in dose with the largest difference in PTV (P) dose of <1.5% in D98% and D2% in all cases (PO and PPLN). Five and six fold increase in calculation times were observed for PO and PPLN plans, respectively, when using 1.25mm grid size as opposed to 2.5mm.

Supplementary table1:- PTV (P) near minimum and near maximum dose and plan calculation time when using 2.5mm vs 1.25mm calculation grid size, all values presented as population Mean ± SD.

|  | 2.5mm | 1.25mm |
| --- | --- | --- |
| **P**O |  |  |
| D_98%_(Gy) | 35.7 ± 0.5 | 36.2 ± 0.5 |
| D_2%_(Gy) | 42.0 ± 0.2 | 42.4 ± 0.4 |
| Time (min) | 1.9 ± 0.3 | 9.1 ±2.0 |
| **PPLN** |  |  |
| D_98%_(Gy) | 35.6 ± 0.3 | 35.9 ± 0.4 |
| D_2%_(Gy) | 42.0 ± 0.3 | 42.3 ± 0.3 |
| Time (min) | 7.5 ± 0.7 | 44.8 ± 5.3 |

**Additional document, Appendix III:**

**All 10F partial arcs PO plans (30 plans representing high dose region), were delivered to PTW Octavius Detector 1000 SRS within the Octavius 4D. The detector size is 2.3×2.3×0.5mm (volume = 0.003 cm^3^). The detector spacing in the inner area (maximum field size = 5.5×5.5 cm) is 2.5 mm center-to-center and in the outer area is 5 mm center-to-center (maximum field size = 10×10 cm). Gamma analysis results are shown in additional figure 5.**

**Moreover, for one patient (for verification purpose) 10F and 6F partial arc plans PO and PPLN plans were delivered to Gafchromic EBT3 films placed in a solid water phantom at 8 cm depth and 11 cm back scatter. Films were then scanned using an Epson Expression 10000XL scanner and analysed using FilmQAPro software (Ashland Inc), using the red channel [2,3]. Gamma analysis was then performed on are presented in Supplementary table 2.**

Table2:- Gamma passing rate for the six plans delivered on Gafchromic EBT3 films (10% low dose threshold)

|  | Pass Rates (%) | | |
| --- | --- | --- | --- |
|  | 3%/3mm | 2%/2mm | 2%/1mm |
| **PO** |  |  |  |
| PO 10F | 100.00 | 99.31 | 94.17 |
| PO 6F | 99.95 | 98.68 | 90.43 |
| PO 6MV | 99.98 | 99.56 | 97.48 |
| **PPLN** |  |  |  |
| PPLN 10F | 99.9 | 99.55 | 97.06 |
| PPLN 6F | 99.57 | 97.27 | 90.55 |
| PPLN 6X | 100.00 | 99.37 | 90.18 |

**References:**

**[1] Stanley DN, Popp T, Ha CS et al. Dosimetric effect of photon beam energy on volumetric modulated arc therapy treatment plan quality due to body habitus in advanced prostate cancer. Pract Radiat Oncol 2015;5(6):e625–e633.**

**[2] Lewis D, Micke A, Yu X, Chan MF. An efficient protocol for radiochromic film dosimetry combining calibration and measurement in a single scan. Med Phys. 2012 Oct;39(10):6339-50. doi: 10.1118/1.4754797.**

**[3] Niroomand-Rad A, Blackwell CR, Coursey BM, Gall KP, Galvin JM, McLaughlin WL, Meigooni AS, Nath R, Rodgers JE, Soares CG. Radiochromic film dosimetry: recommendations of AAPM Radiation Therapy Committee Task Group 55. American Association of Physicists in Medicine. Med Phys. 1998 Nov;25(11):2093-115.**
