# Supplementary figures and images for: Class solutions for SABR-VMAT for high-risk prostate cancer with and without elective nodal irradiation
Source: Radiat Oncol. 2016 Nov 24;11:155. doi: 10.1186/s13014-016-0730-7 (PMC5121961; doi:10.1186/s13014-016-0730-7)

***(a) Prostate only***

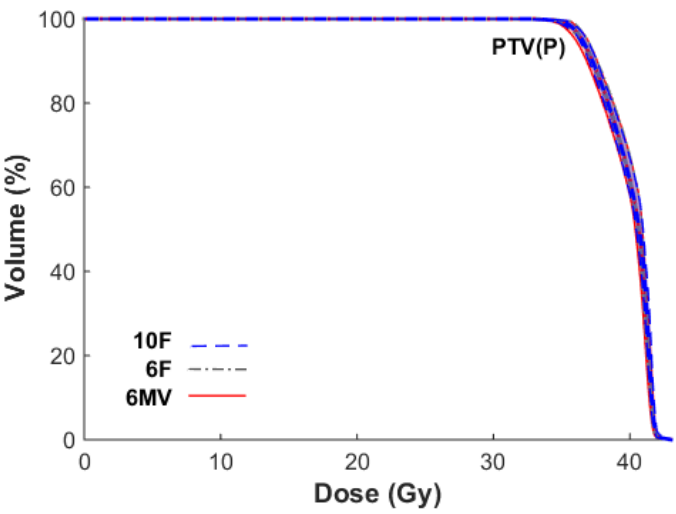

***(b) Prostate and pelvic node***

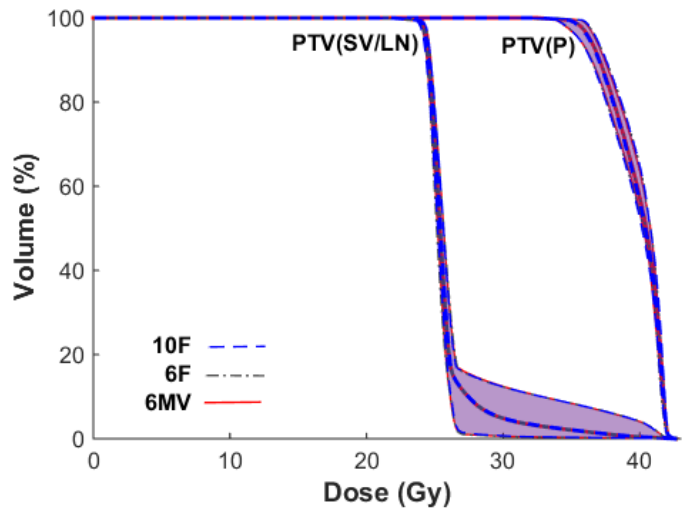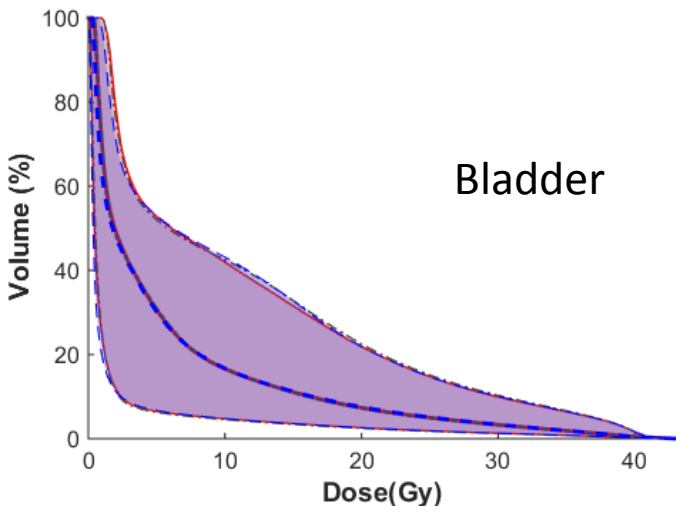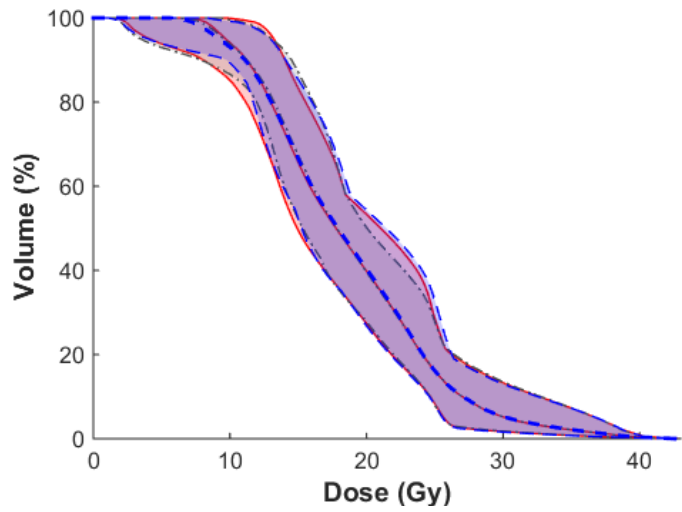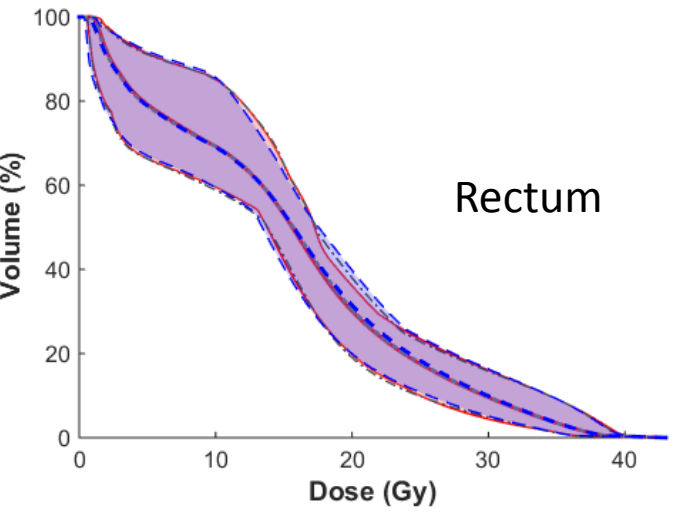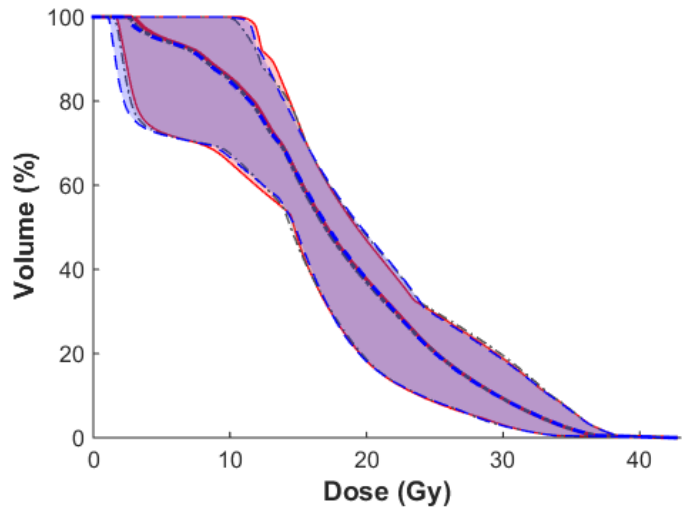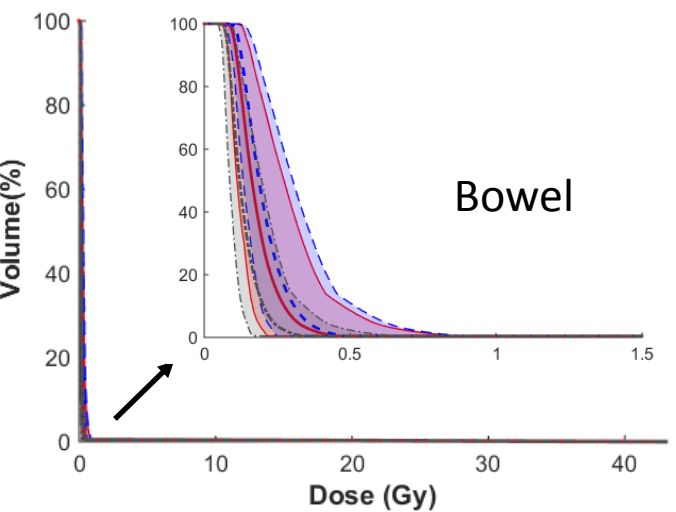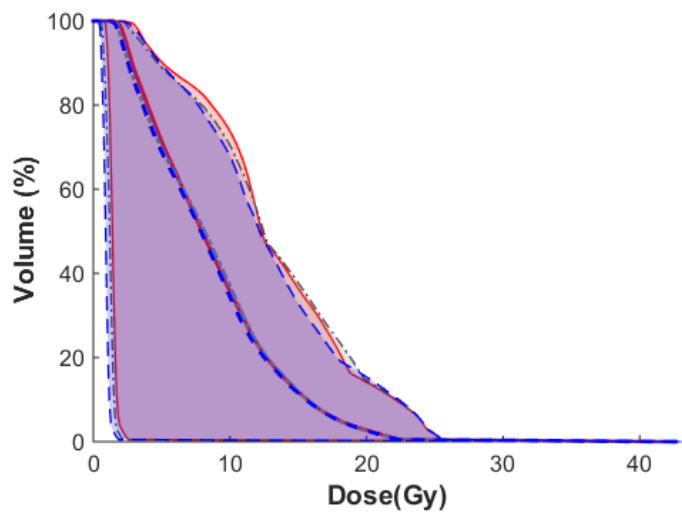

Supplement: Additional file 2: Figure S1. — Population mean dose-volume histograms (DVHs) and standard deviation (shaded areas) for selected structures for full arc (FA) plans using different energies. Left panel: prostate only (PO) single FA plans. Right panel: prostate and pelvic nodes (PPLN) dual full arc (2FA) plans. (PDF 350 kb) [file 13014_2016_730_MOESM2_ESM.pdf]
